# Pes Anserinus Bursitis: A Case Report

**DOI:** 10.7759/cureus.31354

**Published:** 2022-11-11

**Authors:** Michael F Allen, Deborah E Allen

**Affiliations:** 1 Sports Medicine, Royal United Hospital, Bath, GBR; 2 Sports Medicine, Hull York Medical School, York, GBR

**Keywords:** knee pain, tendinopathy, bursitis, pes anserinus syndrome, pes anserinus

## Abstract

Pes anserinus bursitis is a differential diagnosis for knee pain, that may be misdiagnosed. Without proper physical examination and thorough history taking, the diagnosis of pes anserinus may be delayed. We present a case report of this condition, involving both primary care and the emergency department. This case illustrates one possible presentation of this condition, and also demonstrates the risks of overreliance on imaging modalities in primary care, without also performing a proper physical examination of musculoskeletal presentations. The teamwork between physiotherapists and clinicians, in this case, highlights the value of a multidisciplinary team in sports medicine. This case report lends evidence that pes anserinus bursitis should be considered as a possible diagnosis for knee pain and emphasises the importance of physical examination.

## Introduction

The pes anserinus is formed by conjoining three tendons from the thigh muscles that attach to the medial tibia: sartorius, semitendinosus and gracilis. The name, pes anserinus, translates literally from Latin as “goose’s foot” owing to the similarity in shape that the conjoining tendons form as they attach onto the tibia. Within the pes anserinus is a bursa, which sits deep to the pes anserinus tendons, between them and the semimembranosus tendon [1].

The clinical presentation of pes anserinus bursitis usually comprises pain on the medial aspect of the knee, exacerbated by exertion through running or climbing stairs [2]. Associated symptoms may include effusion, or tenderness on palpation, of the region surrounding the bursa; additionally, patients may present with an altered gait, muscle wasting, and reduced function of the knee joint [2].

## Case presentation

A 57-year-old recently-retired lady with a history of mild hypertension and body mass index > 30 kg/m2 presented to the emergency department with an eight-week history of right knee pain. She reported that the pain started following a low-trauma injury: one which occurs suddenly with little force applied. In this instance, the patient’s husband lent on her extended knee and she felt a sharp pain initially that settled into a dull ache, which gradually worsened. This is a typical mechanism of injury for pes anserinus bursitis, whereby direct trauma may predispose to the accumulation of synovial fluid in the bursa, causing inflammation. She was able to fully weight bear immediately but reported an obvious limp.

As the pain, on the medial aspect of the knee, with an aching character, worsened over the next few days, she made an appointment with her general practitioner (GP). The GP took a history of the mechanism of injury and a description of the pain; however, no physical examination was performed at this time. She was booked for a knee MRI; no X-ray was requested since ligament injuries were suspected. The scan reported mild osteoarthritic changes within the knee, which may have contributed to her ongoing symptoms; however, no obvious cause was identified for the acute exacerbation of knee pain. She was then advised to rest and allow the knee to get better with time.

At eight weeks, she attended the ED reporting that her knee pain had neither improved nor worsened in severity, remaining a 7/10 on mobilisation despite this period of rest. The patient was reluctant to return to her GP, wishing to be assessed in secondary care instead. On examination, she was generally physically deconditioned and overweight with a noticeable antalgic gait. She had a full range of motion of her right knee and no noticeable swelling or deformity. Her ligaments all appeared intact and there was no joint line tenderness. On palpation, there was marked point tenderness over the proximal medial tibia with some mild swelling. She was diagnosed clinically with pes anserinus bursitis and was seen immediately by the department physiotherapists. She was advised to use ice and a short course of breakthrough analgesia, whilst undertaking a focused exercise programme using TheraBand. The therapy protocol focused on strengthening and lengthening the muscles of the leg, with a particular focus on quadricep, hamstring, and groin exercises. She had no further investigations and was referred for follow-up with outpatient physiotherapy in six weeks. At follow-up, she reported an improvement in her symptoms, with a reduced limp and improved strength throughout, and attributed this to good compliance with rehabilitation exercises. 

## Discussion

This case report illustrates the value of a multi-disciplinary approach to healthcare, with physiotherapists available in the ED to support clinicians with advice regarding ongoing therapy. One limitation of this case report is that it captures the patient’s condition at a single time-point in their journey through ED, and does not follow them up to the resolution of their condition. Due to the patient’s subsequent referral to primary care, it was not possible to follow up on the patient’s condition beyond this presentation.

Musculoskeletal issues are commonplace within general practice, but this case illustrates how the lack of time that GPs have to undertake consultations can lead to mismanagement of musculoskeletal conditions; this finding is consistent with wider research [3,4]. GPs in the United Kingdom are widely reported to be overstretched and limited in their clinical time. However, this case highlights the importance of ensuring a physical examination is performed in primary care. This case also demonstrates how some clinicians may have an overreliance on imaging modalities, as they are often able to give more definitive information than a clinical examination. However, if the patient is not examined, then it is difficult to give an accurate description within the imaging request. This information is used to specifically guide the reporting radiologist imaging. In this case, it appears that due to the absence of an examination, an MRI of the knee joint was requested due to the presenting complaint of “pain in the knee”. Therefore, the knee MRI and the report of the imaging focused around the knee joint. In this specific case, since the apparent source of the pain is the pes anserinus, which is located inferiorly to the knee joint, there was no comment on the structure and integrity of the pes anserinus by the reporting clinician.

Treatment options for pes anserinus initially include rest and analgesia with non-steroidal anti-inflammatories. Second-line treatment is injection of the pes anserinus bursa with either corticosteroids, anaesthesia, or both [5]. Finally, if medical management fails, surgical resolution of the condition may be achieved through incision and drainage [2].

## Conclusions

Pes anserinus bursitis is frequently misdiagnosed and often not considered a differential diagnosis for a painful knee. This case has highlighted the importance of performing a thorough history and clinical examination when considering the challenge of diagnosing musculoskeletal knee pain, particularly in primary care.

This case has also demonstrated the dangers of overreliance on imaging modalities. In particular, referring patients for imaging too early may lead to a loss of patient confidence and worse patient outcomes, as some musculoskeletal problems do not improve with time alone. 
